# Probing color coherence effects in pp collisions at $\sqrt{s}=7\,\text {TeV}$

**DOI:** 10.1140/epjc/s10052-014-2901-8

**Published:** 2014-06-11

**Authors:** The CMS Collaboration, S. Chatrchyan, V. Khachatryan, A. M. Sirunyan, A. Tumasyan, W. Adam, T. Bergauer, M. Dragicevic, J. Erö, C. Fabjan, M. Friedl, R. Frühwirth, V. M. Ghete, N. Hörmann, J. Hrubec, M. Jeitler, W. Kiesenhofer, V. Knünz, M. Krammer, I. Krätschmer, D. Liko, I. Mikulec, D. Rabady, B. Rahbaran, C. Rohringer, H. Rohringer, R. Schöfbeck, J. Strauss, A. Taurok, W. Treberer-Treberspurg, W. Waltenberger, C.-E. Wulz, V. Mossolov, N. Shumeiko, J. Suarez Gonzalez, S. Alderweireldt, M. Bansal, S. Bansal, T. Cornelis, E. A. De Wolf, X. Janssen, A. Knutsson, S. Luyckx, L. Mucibello, S. Ochesanu, B. Roland, R. Rougny, Z. Staykova, H. Van Haevermaet, P. Van Mechelen, N. Van Remortel, A. Van Spilbeeck, F. Blekman, S. Blyweert, J. D’Hondt, A. Kalogeropoulos, J. Keaveney, S. Lowette, M. Maes, A. Olbrechts, S. Tavernier, W. Van Doninck, P. Van Mulders, G. P. Van Onsem, I. Villella, C. Caillol, B. Clerbaux, G. De Lentdecker, L. Favart, A. P. R. Gay, T. Hreus, A. Léonard, P. E. Marage, A. Mohammadi, L. Perniè, T. Reis, T. Seva, L. Thomas, C. Vander Velde, P. Vanlaer, J. Wang, V. Adler, K. Beernaert, L. Benucci, A. Cimmino, S. Costantini, S. Dildick, G. Garcia, B. Klein, J. Lellouch, A. Marinov, J. Mccartin, A. A. Ocampo Rios, D. Ryckbosch, M. Sigamani, N. Strobbe, F. Thyssen, M. Tytgat, S. Walsh, E. Yazgan, N. Zaganidis, S. Basegmez, C. Beluffi, G. Bruno, R. Castello, A. Caudron, L. Ceard, G. G. Da Silveira, C. Delaere, T. du Pree, D. Favart, L. Forthomme, A. Giammanco, J. Hollar, P. Jez, V. Lemaitre, J. Liao, O. Militaru, C. Nuttens, D. Pagano, A. Pin, K. Piotrzkowski, A. Popov, M. Selvaggi, M. Vidal Marono, J. M. Vizan Garcia, N. Beliy, T. Caebergs, E. Daubie, G. H. Hammad, G. A. Alves, M. Correa Martins Junior, T. Martins, M. E. Pol, M. H. G. Souza, W. L. Aldá Júnior, W. Carvalho, J. Chinellato, A. Custódio, E. M. Da Costa, D. De Jesus Damiao, C. De Oliveira Martins, S. Fonseca De Souza, H. Malbouisson, M. Malek, D. Matos Figueiredo, L. Mundim, H. Nogima, W. L. Prado Da Silva, A. Santoro, A. Sznajder, E. J. Tonelli Manganote, A. Vilela Pereira, F. A. Dias, T. R. Fernandez Perez Tomei, C. Lagana, S. F. Novaes, Sandra S. Padula, C. A. Bernardes, E. M. Gregores, P. G. Mercadante, V. Genchev, P. Iaydjiev, S. Piperov, M. Rodozov, G. Sultanov, M. Vutova, A. Dimitrov, R. Hadjiiska, V. Kozhuharov, L. Litov, B. Pavlov, P. Petkov, J. G. Bian, G. M. Chen, H. S. Chen, C. H. Jiang, D. Liang, S. Liang, X. Meng, J. Tao, X. Wang, Z. Wang, C. Asawatangtrakuldee, Y. Ban, Y. Guo, Q. Li, W. Li, S. Liu, Y. Mao, S. J. Qian, D. Wang, L. Zhang, W. Zou, C. Avila, C. A. Carrillo Montoya, L. F. Chaparro Sierra, J. P. Gomez, B. Gomez Moreno, J. C. Sanabria, N. Godinovic, D. Lelas, R. Plestina, D. Polic, I. Puljak, Z. Antunovic, M. Kovac, V. Brigljevic, K. Kadija, J. Luetic, D. Mekterovic, S. Morovic, L. Tikvica, A. Attikis, G. Mavromanolakis, J. Mousa, C. Nicolaou, F. Ptochos, P. A. Razis, M. Finger, M. Finger, A. A. Abdelalim, Y. Assran, S. Elgammal, A. Ellithi Kamel, M. A. Mahmoud, A. Radi, M. Kadastik, M. Müntel, M. Murumaa, M. Raidal, L. Rebane, A. Tiko, P. Eerola, G. Fedi, M. Voutilainen, J. Härkönen, V. Karimäki, R. Kinnunen, M. J. Kortelainen, T. Lampén, K. Lassila-Perini, S. Lehti, T. Lindén, P. Luukka, T. Mäenpää, T. Peltola, E. Tuominen, J. Tuominiemi, E. Tuovinen, L. Wendland, T. Tuuva, M. Besancon, F. Couderc, M. Dejardin, D. Denegri, B. Fabbro, J. L. Faure, F. Ferri, S. Ganjour, A. Givernaud, P. Gras, G. Hamel de Monchenault, P. Jarry, E. Locci, J. Malcles, L. Millischer, A. Nayak, J. Rander, A. Rosowsky, M. Titov, S. Baffioni, F. Beaudette, L. Benhabib, M. Bluj, P. Busson, C. Charlot, N. Daci, T. Dahms, M. Dalchenko, L. Dobrzynski, A. Florent, R. Granier de Cassagnac, M. Haguenauer, P. Miné, C. Mironov, I. N. Naranjo, M. Nguyen, C. Ochando, P. Paganini, D. Sabes, R. Salerno, Y. Sirois, C. Veelken, A. Zabi, J.-L. Agram, J. Andrea, D. Bloch, J.-M. Brom, E. C. Chabert, C. Collard, E. Conte, F. Drouhin, J.-C. Fontaine, D. Gelé, U. Goerlach, C. Goetzmann, P. Juillot, A.-C. Le Bihan, P. Van Hove, S. Gadrat, S. Beauceron, N. Beaupere, G. Boudoul, S. Brochet, J. Chasserat, R. Chierici, D. Contardo, P. Depasse, H. El Mamouni, J. Fan, J. Fay, S. Gascon, M. Gouzevitch, B. Ille, T. Kurca, M. Lethuillier, L. Mirabito, S. Perries, L. Sgandurra, V. Sordini, M. Vander Donckt, P. Verdier, S. Viret, H. Xiao, Z. Tsamalaidze, C. Autermann, S. Beranek, M. Bontenackels, B. Calpas, M. Edelhoff, L. Feld, N. Heracleous, O. Hindrichs, K. Klein, A. Ostapchuk, A. Perieanu, F. Raupach, J. Sammet, S. Schael, D. Sprenger, H. Weber, B. Wittmer, V. Zhukov, M. Ata, J. Caudron, E. Dietz-Laursonn, D. Duchardt, M. Erdmann, R. Fischer, A. Güth, T. Hebbeker, C. Heidemann, K. Hoepfner, D. Klingebiel, S. Knutzen, P. Kreuzer, M. Merschmeyer, A. Meyer, M. Olschewski, K. Padeken, P. Papacz, H. Pieta, H. Reithler, S. A. Schmitz, L. Sonnenschein, J. Steggemann, D. Teyssier, S. Thüer, M. Weber, V. Cherepanov, Y. Erdogan, G. Flügge, H. Geenen, M. Geisler, W. Haj Ahmad, F. Hoehle, B. Kargoll, T. Kress, Y. Kuessel, J. Lingemann, A. Nowack, I. M. Nugent, L. Perchalla, O. Pooth, A. Stahl, I. Asin, N. Bartosik, J. Behr, W. Behrenhoff, U. Behrens, A. J. Bell, M. Bergholz, A. Bethani, K. Borras, A. Burgmeier, A. Cakir, L. Calligaris, A. Campbell, S. Choudhury, F. Costanza, C. Diez Pardos, S. Dooling, T. Dorland, G. Eckerlin, D. Eckstein, G. Flucke, A. Geiser, I. Glushkov, A. Grebenyuk, P. Gunnellini, S. Habib, J. Hauk, G. Hellwig, D. Horton, H. Jung, M. Kasemann, P. Katsas, C. Kleinwort, H. Kluge, M. Krämer, D. Krücker, E. Kuznetsova, W. Lange, J. Leonard, K. Lipka, W. Lohmann, B. Lutz, R. Mankel, I. Marfin, I.-A. Melzer-Pellmann, A. B. Meyer, J. Mnich, A. Mussgiller, S. Naumann-Emme, O. Novgorodova, F. Nowak, J. Olzem, H. Perrey, A. Petrukhin, D. Pitzl, R. Placakyte, A. Raspereza, P. M. Ribeiro Cipriano, C. Riedl, E. Ron, M. Ö. Sahin, J. Salfeld-Nebgen, R. Schmidt, T. Schoerner-Sadenius, N. Sen, M. Stein, R. Walsh, C. Wissing, M. Aldaya Martin, V. Blobel, H. Enderle, J. Erfle, E. Garutti, U. Gebbert, M. Görner, M. Gosselink, J. Haller, K. Heine, R. S. Höing, G. Kaussen, H. Kirschenmann, R. Klanner, R. Kogler, J. Lange, I. Marchesini, T. Peiffer, N. Pietsch, D. Rathjens, C. Sander, H. Schettler, P. Schleper, E. Schlieckau, A. Schmidt, M. Schröder, T. Schum, M. Seidel, J. Sibille, V. Sola, H. Stadie, G. Steinbrück, J. Thomsen, D. Troendle, E. Usai, L. Vanelderen, C. Barth, C. Baus, J. Berger, C. Böser, E. Butz, T. Chwalek, W. De Boer, A. Descroix, A. Dierlamm, M. Feindt, M. Guthoff, F. Hartmann, T. Hauth, H. Held, K. H. Hoffmann, U. Husemann, I. Katkov, J. R. Komaragiri, A. Kornmayer, P. Lobelle Pardo, D. Martschei, M. U. Mozer, Th. Müller, M. Niegel, A. Nürnberg, O. Oberst, J. Ott, G. Quast, K. Rabbertz, F. Ratnikov, S. Röcker, F.-P. Schilling, G. Schott, H. J. Simonis, F. M. Stober, R. Ulrich, J. Wagner-Kuhr, S. Wayand, T. Weiler, M. Zeise, G. Anagnostou, G. Daskalakis, T. Geralis, S. Kesisoglou, A. Kyriakis, D. Loukas, A. Markou, C. Markou, E. Ntomari, I. Topsis-giotis, L. Gouskos, A. Panagiotou, N. Saoulidou, E. Stiliaris, X. Aslanoglou, I. Evangelou, G. Flouris, C. Foudas, P. Kokkas, N. Manthos, I. Papadopoulos, E. Paradas, G. Bencze, C. Hajdu, P. Hidas, D. Horvath, F. Sikler, V. Veszpremi, G. Vesztergombi, A. J. Zsigmond, N. Beni, S. Czellar, J. Molnar, J. Palinkas, Z. Szillasi, J. Karancsi, P. Raics, Z. L. Trocsanyi, B. Ujvari, S. K. Swain, S. B. Beri, V. Bhatnagar, N. Dhingra, R. Gupta, M. Kaur, M. Z. Mehta, M. Mittal, N. Nishu, A. Sharma, J. B. Singh, Ashok Kumar, Arun Kumar, S. Ahuja, A. Bhardwaj, B. C. Choudhary, A. Kumar, S. Malhotra, M. Naimuddin, K. Ranjan, P. Saxena, V. Sharma, R. K. Shivpuri, S. Banerjee, S. Bhattacharya, K. Chatterjee, S. Dutta, B. Gomber, Sa. Jain, Sh. Jain, R. Khurana, A. Modak, S. Mukherjee, D. Roy, S. Sarkar, M. Sharan, A. P. Singh, A. Abdulsalam, D. Dutta, S. Kailas, V. Kumar, A. K. Mohanty, L. M. Pant, P. Shukla, A. Topkar, T. Aziz, R. M. Chatterjee, S. Ganguly, S. Ghosh, M. Guchait, A. Gurtu, G. Kole, S. Kumar, M. Maity, G. Majumder, K. Mazumdar, G. B. Mohanty, B. Parida, K. Sudhakar, N. Wickramage, S. Dugad, H. Arfaei, H. Bakhshiansohi, S. M. Etesami, A. Fahim, A. Jafari, M. Khakzad, M. Mohammadi Najafabadi, S. Paktinat Mehdiabadi, B. Safarzadeh, M. Zeinali, M. Grunewald, M. Abbrescia, L. Barbone, C. Calabria, S. S. Chhibra, A. Colaleo, D. Creanza, N. De Filippis, M. De Palma, L. Fiore, G. Iaselli, G. Maggi, M. Maggi, B. Marangelli, S. My, S. Nuzzo, N. Pacifico, A. Pompili, G. Pugliese, G. Selvaggi, L. Silvestris, G. Singh, R. Venditti, P. Verwilligen, G. Zito, G. Abbiendi, A. C. Benvenuti, D. Bonacorsi, S. Braibant-Giacomelli, L. Brigliadori, R. Campanini, P. Capiluppi, A. Castro, F. R. Cavallo, G. Codispoti, M. Cuffiani, G. M. Dallavalle, F. Fabbri, A. Fanfani, D. Fasanella, P. Giacomelli, C. Grandi, L. Guiducci, S. Marcellini, G. Masetti, M. Meneghelli, A. Montanari, F. L. Navarria, F. Odorici, A. Perrotta, F. Primavera, A. M. Rossi, T. Rovelli, G. P. Siroli, N. Tosi, R. Travaglini, S. Albergo, G. Cappello, M. Chiorboli, S. Costa, F. Giordano, R. Potenza, A. Tricomi, C. Tuve, G. Barbagli, V. Ciulli, C. Civinini, R. D’Alessandro, E. Focardi, S. Frosali, E. Gallo, S. Gonzi, V. Gori, P. Lenzi, M. Meschini, S. Paoletti, G. Sguazzoni, A. Tropiano, L. Benussi, S. Bianco, F. Fabbri, D. Piccolo, P. Fabbricatore, R. Ferretti, F. Ferro, M. Lo Vetere, R. Musenich, E. Robutti, S. Tosi, A. Benaglia, M. E. Dinardo, S. Fiorendi, S. Gennai, A. Ghezzi, P. Govoni, M. T. Lucchini, S. Malvezzi, R. A. Manzoni, A. Martelli, D. Menasce, L. Moroni, M. Paganoni, D. Pedrini, S. Ragazzi, N. Redaelli, T. Tabarelli de Fatis, S. Buontempo, N. Cavallo, A. De Cosa, F. Fabozzi, A. O. M. Iorio, L. Lista, S. Meola, M. Merola, P. Paolucci, P. Azzi, N. Bacchetta, M. Bellato, D. Bisello, A. Branca, R. Carlin, P. Checchia, T. Dorigo, U. Dosselli, M. Galanti, F. Gasparini, U. Gasparini, P. Giubilato, A. Gozzelino, K. Kanishchev, S. Lacaprara, I. Lazzizzera, M. Margoni, A. T. Meneguzzo, J. Pazzini, N. Pozzobon, P. Ronchese, M. Sgaravatto, F. Simonetto, E. Torassa, M. Tosi, A. Triossi, P. Zotto, A. Zucchetta, G. Zumerle, M. Gabusi, S. P. Ratti, C. Riccardi, P. Vitulo, M. Biasini, G. M. Bilei, L. Fanò, P. Lariccia, G. Mantovani, M. Menichelli, A. Nappi, F. Romeo, A. Saha, A. Santocchia, A. Spiezia, K. Androsov, P. Azzurri, G. Bagliesi, J. Bernardini, T. Boccali, G. Broccolo, R. Castaldi, M. A. Ciocci, R. T. D’Agnolo, R. Dell’Orso, F. Fiori, L. Foà, A. Giassi, M. T. Grippo, A. Kraan, F. Ligabue, T. Lomtadze, L. Martini, A. Messineo, C. S. Moon, F. Palla, A. Rizzi, A. Savoy-Navarro, A. T. Serban, P. Spagnolo, P. Squillacioti, R. Tenchini, G. Tonelli, A. Venturi, P. G. Verdini, C. Vernieri, L. Barone, F. Cavallari, D. Del Re, M. Diemoz, M. Grassi, E. Longo, F. Margaroli, P. Meridiani, F. Micheli, S. Nourbakhsh, G. Organtini, R. Paramatti, S. Rahatlou, C. Rovelli, L. Soffi, N. Amapane, R. Arcidiacono, S. Argiro, M. Arneodo, R. Bellan, C. Biino, N. Cartiglia, S. Casasso, M. Costa, A. Degano, N. Demaria, C. Mariotti, S. Maselli, E. Migliore, V. Monaco, M. Musich, M. M. Obertino, N. Pastrone, M. Pelliccioni, A. Potenza, A. Romero, M. Ruspa, R. Sacchi, A. Solano, A. Staiano, U. Tamponi, S. Belforte, V. Candelise, M. Casarsa, F. Cossutti, G. Della Ricca, B. Gobbo, C. La Licata, M. Marone, D. Montanino, A. Penzo, A. Schizzi, A. Zanetti, S. Chang, T. Y. Kim, S. K. Nam, D. H. Kim, G. N. Kim, J. E. Kim, D. J. Kong, S. Lee, Y. D. Oh, H. Park, D. C. Son, J. Y. Kim, Zero J. Kim, S. Song, S. Choi, D. Gyun, B. Hong, M. Jo, H. Kim, T. J. Kim, K. S. Lee, S. K. Park, Y. Roh, M. Choi, J. H. Kim, C. Park, I. C. Park, S. Park, G. Ryu, Y. Choi, Y. K. Choi, J. Goh, M. S. Kim, E. Kwon, B. Lee, J. Lee, H. Seo, I. Yu, I. Grigelionis, A. Juodagalvis, H. Castilla-Valdez, E. De La Cruz-Burelo, I. Heredia-de La Cruz, R. Lopez-Fernandez, J. Martínez-Ortega, A. Sanchez-Hernandez, L. M. Villasenor-Cendejas, S. Carrillo Moreno, F. Vazquez Valencia, H. A. Salazar Ibarguen, E. Casimiro Linares, A. Morelos Pineda, M. A. Reyes-Santos, D. Krofcheck, P. H. Butler, R. Doesburg, S. Reucroft, H. Silverwood, M. Ahmad, M. I. Asghar, J. Butt, H. R. Hoorani, S. Khalid, W. A. Khan, T. Khurshid, S. Qazi, M. A. Shah, M. Shoaib, H. Bialkowska, B. Boimska, T. Frueboes, M. Górski, M. Kazana, K. Nawrocki, K. Romanowska-Rybinska, M. Szleper, G. Wrochna, P. Zalewski, G. Brona, K. Bunkowski, M. Cwiok, W. Dominik, K. Doroba, A. Kalinowski, M. Konecki, J. Krolikowski, M. Misiura, W. Wolszczak, N. Almeida, P. Bargassa, C. Beirão Da Cruz E. Silva, P. Faccioli, P. G. Ferreira Parracho, M. Gallinaro, F. Nguyen, J. Rodrigues Antunes, J. Seixas, J. Varela, P. Vischia, S. Afanasiev, P. Bunin, M. Gavrilenko, I. Golutvin, I. Gorbunov, A. Kamenev, V. Karjavin, V. Konoplyanikov, A. Lanev, A. Malakhov, V. Matveev, P. Moisenz, V. Palichik, V. Perelygin, S. Shmatov, N. Skatchkov, V. Smirnov, A. Zarubin, S. Evstyukhin, V. Golovtsov, Y. Ivanov, V. Kim, P. Levchenko, V. Murzin, V. Oreshkin, I. Smirnov, V. Sulimov, L. Uvarov, S. Vavilov, A. Vorobyev, An. Vorobyev, Yu. Andreev, A. Dermenev, S. Gninenko, N. Golubev, M. Kirsanov, N. Krasnikov, A. Pashenkov, D. Tlisov, A. Toropin, V. Epshteyn, M. Erofeeva, V. Gavrilov, N. Lychkovskaya, V. Popov, G. Safronov, S. Semenov, A. Spiridonov, V. Stolin, E. Vlasov, A. Zhokin, V. Andreev, M. Azarkin, I. Dremin, M. Kirakosyan, A. Leonidov, G. Mesyats, S. V. Rusakov, A. Vinogradov, E. Boos, M. Dubinin, L. Dudko, A. Ershov, A. Gribushin, V. Klyukhin, O. Kodolova, I. Lokhtin, A. Markina, S. Obraztsov, S. Petrushanko, V. Savrin, A. Snigirev, I. Azhgirey, I. Bayshev, S. Bitioukov, V. Kachanov, A. Kalinin, D. Konstantinov, V. Krychkine, V. Petrov, R. Ryutin, A. Sobol, L. Tourtchanovitch, S. Troshin, N. Tyurin, A. Uzunian, A. Volkov, P. Adzic, M. Djordjevic, M. Ekmedzic, D. Krpic, J. Milosevic, M. Aguilar-Benitez, J. Alcaraz Maestre, C. Battilana, E. Calvo, M. Cerrada, M. Chamizo Llatas, N. Colino, B. De La Cruz, A. Delgado Peris, D. Domínguez Vázquez, C. Fernandez Bedoya, J. P. Fernández Ramos, A. Ferrando, J. Flix, M. C. Fouz, P. Garcia-Abia, O. Gonzalez Lopez, S. Goy Lopez, J. M. Hernandez, M. I. Josa, G. Merino, E. Navarro De Martino, J. Puerta Pelayo, A. Quintario Olmeda, I. Redondo, L. Romero, J. Santaolalla, M. S. Soares, C. Willmott, C. Albajar, J. F. de Trocóniz, H. Brun, J. Cuevas, J. Fernandez Menendez, S. Folgueras, I. Gonzalez Caballero, L. Lloret Iglesias, J. Piedra Gomez, J. A. Brochero Cifuentes, I. J. Cabrillo, A. Calderon, S. H. Chuang, J. Duarte Campderros, M. Fernandez, G. Gomez, J. Gonzalez Sanchez, A. Graziano, C. Jorda, A. Lopez Virto, J. Marco, R. Marco, C. Martinez Rivero, F. Matorras, F. J. Munoz Sanchez, T. Rodrigo, A. Y. Rodríguez-Marrero, A. Ruiz-Jimeno, L. Scodellaro, I. Vila, R. Vilar Cortabitarte, D. Abbaneo, E. Auffray, G. Auzinger, M. Bachtis, P. Baillon, A. H. Ball, D. Barney, J. Bendavid, J. F. Benitez, C. Bernet, G. Bianchi, P. Bloch, A. Bocci, A. Bonato, O. Bondu, C. Botta, H. Breuker, T. Camporesi, G. Cerminara, T. Christiansen, J. A. Coarasa Perez, S. Colafranceschi, M. D’Alfonso, D. d’Enterria, A. Dabrowski, A. David, F. De Guio, A. De Roeck, S. De Visscher, S. Di Guida, M. Dobson, N. Dupont-Sagorin, A. Elliott-Peisert, J. Eugster, G. Franzoni, W. Funk, G. Georgiou, M. Giffels, D. Gigi, K. Gill, D. Giordano, M. Girone, M. Giunta, F. Glege, R. Gomez-Reino Garrido, S. Gowdy, R. Guida, J. Hammer, M. Hansen, P. Harris, C. Hartl, A. Hinzmann, V. Innocente, P. Janot, E. Karavakis, K. Kousouris, K. Krajczar, P. Lecoq, Y.-J. Lee, C. Lourenço, N. Magini, L. Malgeri, M. Mannelli, L. Masetti, F. Meijers, S. Mersi, E. Meschi, R. Moser, M. Mulders, P. Musella, E. Nesvold, L. Orsini, E. Palencia Cortezon, E. Perez, L. Perrozzi, A. Petrilli, A. Pfeiffer, M. Pierini, M. Pimiä, D. Piparo, M. Plagge, L. Quertenmont, A. Racz, W. Reece, G. Rolandi, M. Rovere, H. Sakulin, F. Santanastasio, C. Schäfer, C. Schwick, S. Sekmen, P. Siegrist, P. Silva, M. Simon, P. Sphicas, D. Spiga, B. Stieger, M. Stoye, A. Tsirou, G. I. Veres, J. R. Vlimant, H. K. Wöhri, S. D. Worm, W. D. Zeuner, W. Bertl, K. Deiters, W. Erdmann, K. Gabathuler, R. Horisberger, Q. Ingram, H. C. Kaestli, S. König, D. Kotlinski, U. Langenegger, D. Renker, T. Rohe, F. Bachmair, L. Bäni, L. Bianchini, P. Bortignon, M. A. Buchmann, B. Casal, N. Chanon, A. Deisher, G. Dissertori, M. Dittmar, M. Donegà, M. Dünser, P. Eller, K. Freudenreich, C. Grab, D. Hits, P. Lecomte, W. Lustermann, B. Mangano, A. C. Marini, P. Martinez Ruiz del Arbol, D. Meister, N. Mohr, F. Moortgat, C. Nägeli, P. Nef, F. Nessi-Tedaldi, F. Pandolfi, L. Pape, F. Pauss, M. Peruzzi, M. Quittnat, F. J. Ronga, M. Rossini, L. Sala, A. K. Sanchez, A. Starodumov, M. Takahashi, L. Tauscher, A. Thea, K. Theofilatos, D. Treille, C. Urscheler, R. Wallny, H. A. Weber, C. Amsler, V. Chiochia, C. Favaro, M. Ivova Rikova, B. Kilminster, B. Millan Mejias, P. Otiougova, P. Robmann, H. Snoek, S. Taroni, M. Verzetti, Y. Yang, M. Cardaci, K. H. Chen, C. Ferro, C. M. Kuo, S. W. Li, W. Lin, Y. J. Lu, R. Volpe, S. S. Yu, P. Bartalini, P. Chang, Y. H. Chang, Y. W. Chang, Y. Chao, K. F. Chen, C. Dietz, U. Grundler, W.-S. Hou, Y. Hsiung, K. Y. Kao, Y. J. Lei, R.-S. Lu, D. Majumder, E. Petrakou, X. Shi, J. G. Shiu, Y. M. Tzeng, M. Wang, B. Asavapibhop, N. Suwonjandee, A. Adiguzel, M. N. Bakirci, S. Cerci, C. Dozen, I. Dumanoglu, E. Eskut, S. Girgis, G. Gokbulut, E. Gurpinar, I. Hos, E. E. Kangal, A. Kayis Topaksu, G. Onengut, K. Ozdemir, S. Ozturk, A. Polatoz, K. Sogut, D. Sunar Cerci, B. Tali, H. Topakli, M. Vergili, I. V. Akin, T. Aliev, B. Bilin, S. Bilmis, M. Deniz, H. Gamsizkan, A. M. Guler, G. Karapinar, K. Ocalan, A. Ozpineci, M. Serin, R. Sever, U. E. Surat, M. Yalvac, M. Zeyrek, E. Gülmez, B. Isildak, M. Kaya, O. Kaya, S. Ozkorucuklu, N. Sonmez, H. Bahtiyar, E. Barlas, K. Cankocak, Y. O. Günaydin, F. I. Vardarlı, M. Yücel, L. Levchuk, P. Sorokin, J. J. Brooke, E. Clement, D. Cussans, H. Flacher, R. Frazier, J. Goldstein, M. Grimes, G. P. Heath, H. F. Heath, L. Kreczko, C. Lucas, Z. Meng, S. Metson, D. M. Newbold, K. Nirunpong, S. Paramesvaran, A. Poll, S. Senkin, V. J. Smith, T. Williams, K. W. Bell, A. Belyaev, C. Brew, R. M. Brown, D. J. A. Cockerill, J. A. Coughlan, K. Harder, S. Harper, J. Ilic, E. Olaiya, D. Petyt, B. C. Radburn-Smith, C. H. Shepherd-Themistocleous, I. R. Tomalin, W. J. Womersley, R. Bainbridge, O. Buchmuller, D. Burton, D. Colling, N. Cripps, M. Cutajar, P. Dauncey, G. Davies, M. Della Negra, W. Ferguson, J. Fulcher, D. Futyan, A. Gilbert, A. Guneratne Bryer, G. Hall, Z. Hatherell, J. Hays, G. Iles, M. Jarvis, G. Karapostoli, M. Kenzie, R. Lane, R. Lucas, L. Lyons, A.-M. Magnan, J. Marrouche, B. Mathias, R. Nandi, J. Nash, A. Nikitenko, J. Pela, M. Pesaresi, K. Petridis, M. Pioppi, D. M. Raymond, S. Rogerson, A. Rose, C. Seez, P. Sharp, A. Sparrow, A. Tapper, M. Vazquez Acosta, T. Virdee, S. Wakefield, N. Wardle, M. Chadwick, J. E. Cole, P. R. Hobson, A. Khan, P. Kyberd, D. Leggat, D. Leslie, W. Martin, I. D. Reid, P. Symonds, L. Teodorescu, M. Turner, J. Dittmann, K. Hatakeyama, A. Kasmi, H. Liu, T. Scarborough, O. Charaf, S. I. Cooper, C. Henderson, P. Rumerio, A. Avetisyan, T. Bose, C. Fantasia, A. Heister, P. Lawson, D. Lazic, J. Rohlf, D. Sperka, J. St. John, L. Sulak, J. Alimena, G. Christopher, D. Cutts, Z. Demiragli, A. Ferapontov, A. Garabedian, U. Heintz, S. Jabeen, G. Kukartsev, E. Laird, G. Landsberg, M. Luk, M. Narain, M. Segala, T. Sinthuprasith, T. Speer, R. Breedon, G. Breto, M. Calderon De La Barca Sanchez, S. Chauhan, M. Chertok, J. Conway, R. Conway, P. T. Cox, R. Erbacher, M. Gardner, R. Houtz, W. Ko, A. Kopecky, R. Lander, T. Miceli, D. Pellett, J. Pilot, F. Ricci-Tam, B. Rutherford, M. Searle, S. Shalhout, J. Smith, M. Squires, M. Tripathi, S. Wilbur, R. Yohay, V. Andreev, D. Cline, R. Cousins, S. Erhan, P. Everaerts, C. Farrell, M. Felcini, J. Hauser, M. Ignatenko, C. Jarvis, G. Rakness, P. Schlein, E. Takasugi, P. Traczyk, V. Valuev, J. Babb, R. Clare, J. Ellison, J. W. Gary, G. Hanson, J. Heilman, P. Jandir, H. Liu, O. R. Long, A. Luthra, M. Malberti, H. Nguyen, A. Shrinivas, J. Sturdy, S. Sumowidagdo, R. Wilken, S. Wimpenny, W. Andrews, J. G. Branson, G. B. Cerati, S. Cittolin, D. Evans, A. Holzner, R. Kelley, M. Lebourgeois, J. Letts, I. Macneill, S. Padhi, C. Palmer, G. Petrucciani, M. Pieri, M. Sani, S. Simon, E. Sudano, M. Tadel, Y. Tu, A. Vartak, S. Wasserbaech, F. Würthwein, A. Yagil, J. Yoo, D. Barge, C. Campagnari, T. Danielson, K. Flowers, P. Geffert, C. George, F. Golf, J. Incandela, C. Justus, D. Kovalskyi, V. Krutelyov, R. Magaña Villalba, N. Mccoll, V. Pavlunin, J. Richman, R. Rossin, D. Stuart, W. To, C. West, A. Apresyan, A. Bornheim, J. Bunn, Y. Chen, E. Di Marco, J. Duarte, D. Kcira, Y. Ma, A. Mott, H. B. Newman, C. Pena, C. Rogan, M. Spiropulu, V. Timciuc, J. Veverka, R. Wilkinson, S. Xie, R. Y. Zhu, V. Azzolini, A. Calamba, R. Carroll, T. Ferguson, Y. Iiyama, D. W. Jang, Y. F. Liu, M. Paulini, J. Russ, H. Vogel, I. Vorobiev, J. P. Cumalat, B. R. Drell, W. T. Ford, A. Gaz, E. Luiggi Lopez, U. Nauenberg, J. G. Smith, K. Stenson, K. A. Ulmer, S. R. Wagner, J. Alexander, A. Chatterjee, N. Eggert, L. K. Gibbons, W. Hopkins, A. Khukhunaishvili, B. Kreis, N. Mirman, G. Nicolas Kaufman, J. R. Patterson, A. Ryd, E. Salvati, W. Sun, W. D. Teo, J. Thom, J. Thompson, J. Tucker, Y. Weng, L. Winstrom, P. Wittich, D. Winn, S. Abdullin, M. Albrow, J. Anderson, G. Apollinari, L. A. T. Bauerdick, A. Beretvas, J. Berryhill, P. C. Bhat, K. Burkett, J. N. Butler, V. Chetluru, H. W. K. Cheung, F. Chlebana, S. Cihangir, V. D. Elvira, I. Fisk, J. Freeman, Y. Gao, E. Gottschalk, L. Gray, D. Green, O. Gutsche, D. Hare, R. M. Harris, J. Hirschauer, B. Hooberman, S. Jindariani, M. Johnson, U. Joshi, K. Kaadze, B. Klima, S. Kunori, S. Kwan, J. Linacre, D. Lincoln, R. Lipton, J. Lykken, K. Maeshima, J. M. Marraffino, V. I. Martinez Outschoorn, S. Maruyama, D. Mason, P. McBride, K. Mishra, S. Mrenna, Y. Musienko, C. Newman-Holmes, V. O’Dell, O. Prokofyev, N. Ratnikova, E. Sexton-Kennedy, S. Sharma, W. J. Spalding, L. Spiegel, L. Taylor, S. Tkaczyk, N. V. Tran, L. Uplegger, E. W. Vaandering, R. Vidal, J. Whitmore, W. Wu, F. Yang, J. C. Yun, D. Acosta, P. Avery, D. Bourilkov, M. Chen, T. Cheng, S. Das, M. De Gruttola, G. P. Di Giovanni, D. Dobur, A. Drozdetskiy, R. D. Field, M. Fisher, Y. Fu, I. K. Furic, J. Hugon, B. Kim, J. Konigsberg, A. Korytov, A. Kropivnitskaya, T. Kypreos, J. F. Low, K. Matchev, P. Milenovic, G. Mitselmakher, L. Muniz, R. Remington, A. Rinkevicius, N. Skhirtladze, M. Snowball, J. Yelton, M. Zakaria, V. Gaultney, S. Hewamanage, S. Linn, P. Markowitz, G. Martinez, J. L. Rodriguez, T. Adams, A. Askew, J. Bochenek, J. Chen, B. Diamond, J. Haas, S. Hagopian, V. Hagopian, K. F. Johnson, H. Prosper, V. Veeraraghavan, M. Weinberg, M. M. Baarmand, B. Dorney, M. Hohlmann, H. Kalakhety, F. Yumiceva, M. R. Adams, L. Apanasevich, V. E. Bazterra, R. R. Betts, I. Bucinskaite, J. Callner, R. Cavanaugh, O. Evdokimov, L. Gauthier, C. E. Gerber, D. J. Hofman, S. Khalatyan, P. Kurt, F. Lacroix, D. H. Moon, C. O’Brien, C. Silkworth, D. Strom, P. Turner, N. Varelas, U. Akgun, E. A. Albayrak, B. Bilki, W. Clarida, K. Dilsiz, F. Duru, S. Griffiths, J.-P. Merlo, H. Mermerkaya, A. Mestvirishvili, A. Moeller, J. Nachtman, C. R. Newsom, H. Ogul, Y. Onel, F. Ozok, S. Sen, P. Tan, E. Tiras, J. Wetzel, T. Yetkin, K. Yi, B. A. Barnett, B. Blumenfeld, S. Bolognesi, G. Giurgiu, A. V. Gritsan, G. Hu, P. Maksimovic, C. Martin, M. Swartz, A. Whitbeck, P. Baringer, A. Bean, G. Benelli, R. P. Kenny, M. Murray, D. Noonan, S. Sanders, R. Stringer, J. S. Wood, A. F. Barfuss, I. Chakaberia, A. Ivanov, S. Khalil, M. Makouski, Y. Maravin, L. K. Saini, S. Shrestha, I. Svintradze, J. Gronberg, D. Lange, F. Rebassoo, D. Wright, A. Baden, B. Calvert, S. C. Eno, J. A. Gomez, N. J. Hadley, R. G. Kellogg, T. Kolberg, Y. Lu, M. Marionneau, A. C. Mignerey, K. Pedro, A. Peterman, A. Skuja, J. Temple, M. B. Tonjes, S. C. Tonwar, A. Apyan, G. Bauer, W. Busza, I. A. Cali, M. Chan, L. Di Matteo, V. Dutta, G. Gomez Ceballos, M. Goncharov, D. Gulhan, Y. Kim, M. Klute, Y. S. Lai, A. Levin, P. D. Luckey, T. Ma, S. Nahn, C. Paus, D. Ralph, C. Roland, G. Roland, G. S. F. Stephans, F. Stöckli, K. Sumorok, D. Velicanu, R. Wolf, B. Wyslouch, M. Yang, Y. Yilmaz, A. S. Yoon, M. Zanetti, V. Zhukova, B. Dahmes, A. De Benedetti, A. Gude, J. Haupt, S. C. Kao, K. Klapoetke, Y. Kubota, J. Mans, N. Pastika, R. Rusack, M. Sasseville, A. Singovsky, N. Tambe, J. Turkewitz, J. G. Acosta, L. M. Cremaldi, R. Kroeger, S. Oliveros, L. Perera, R. Rahmat, D. A. Sanders, D. Summers, E. Avdeeva, K. Bloom, S. Bose, D. R. Claes, A. Dominguez, M. Eads, R. Gonzalez Suarez, J. Keller, I. Kravchenko, J. Lazo-Flores, S. Malik, F. Meier, G. R. Snow, J. Dolen, A. Godshalk, I. Iashvili, S. Jain, A. Kharchilava, S. Rappoccio, Z. Wan, G. Alverson, E. Barberis, D. Baumgartel, M. Chasco, J. Haley, A. Massironi, D. Nash, T. Orimoto, D. Trocino, D. Wood, J. Zhang, A. Anastassov, K. A. Hahn, A. Kubik, L. Lusito, N. Mucia, N. Odell, B. Pollack, A. Pozdnyakov, M. Schmitt, S. Stoynev, K. Sung, M. Velasco, S. Won, D. Berry, A. Brinkerhoff, K. M. Chan, M. Hildreth, C. Jessop, D. J. Karmgard, J. Kolb, K. Lannon, W. Luo, S. Lynch, N. Marinelli, D. M. Morse, T. Pearson, M. Planer, R. Ruchti, J. Slaunwhite, N. Valls, M. Wayne, M. Wolf, L. Antonelli, B. Bylsma, L. S. Durkin, C. Hill, R. Hughes, K. Kotov, T. Y. Ling, D. Puigh, M. Rodenburg, G. Smith, C. Vuosalo, B. L. Winer, H. Wolfe, E. Berry, P. Elmer, V. Halyo, P. Hebda, J. Hegeman, A. Hunt, P. Jindal, S. A. Koay, P. Lujan, D. Marlow, T. Medvedeva, M. Mooney, J. Olsen, P. Piroué, X. Quan, A. Raval, H. Saka, D. Stickland, C. Tully, J. S. Werner, S. C. Zenz, A. Zuranski, E. Brownson, A. Lopez, H. Mendez, J. E. Ramirez Vargas, E. Alagoz, D. Benedetti, G. Bolla, D. Bortoletto, M. De Mattia, A. Everett, Z. Hu, M. Jones, K. Jung, O. Koybasi, M. Kress, N. Leonardo, D. Lopes Pegna, V. Maroussov, P. Merkel, D. H. Miller, N. Neumeister, I. Shipsey, D. Silvers, A. Svyatkovskiy, F. Wang, W. Xie, L. Xu, H. D. Yoo, J. Zablocki, Y. Zheng, N. Parashar, A. Adair, B. Akgun, K. M. Ecklund, F. J. M. Geurts, W. Li, B. Michlin, B. P. Padley, R. Redjimi, J. Roberts, J. Zabel, B. Betchart, A. Bodek, R. Covarelli, P. de Barbaro, R. Demina, Y. Eshaq, T. Ferbel, A. Garcia-Bellido, P. Goldenzweig, J. Han, A. Harel, D. C. Miner, G. Petrillo, D. Vishnevskiy, M. Zielinski, A. Bhatti, R. Ciesielski, L. Demortier, K. Goulianos, G. Lungu, S. Malik, C. Mesropian, S. Arora, A. Barker, J. P. Chou, C. Contreras-Campana, E. Contreras-Campana, D. Duggan, D. Ferencek, Y. Gershtein, R. Gray, E. Halkiadakis, D. Hidas, A. Lath, S. Panwalkar, M. Park, R. Patel, V. Rekovic, J. Robles, S. Salur, S. Schnetzer, C. Seitz, S. Somalwar, R. Stone, S. Thomas, P. Thomassen, M. Walker, G. Cerizza, M. Hollingsworth, K. Rose, S. Spanier, Z. C. Yang, A. York, O. Bouhali, R. Eusebi, W. Flanagan, J. Gilmore, T. Kamon, V. Khotilovich, R. Montalvo, I. Osipenkov, Y. Pakhotin, A. Perloff, J. Roe, A. Safonov, T. Sakuma, I. Suarez, A. Tatarinov, D. Toback, N. Akchurin, C. Cowden, J. Damgov, C. Dragoiu, P. R. Dudero, K. Kovitanggoon, S. W. Lee, T. Libeiro, I. Volobouev, E. Appelt, A. G. Delannoy, S. Greene, A. Gurrola, W. Johns, C. Maguire, A. Melo, M. Sharma, P. Sheldon, B. Snook, S. Tuo, J. Velkovska, M. W. Arenton, S. Boutle, B. Cox, B. Francis, J. Goodell, R. Hirosky, A. Ledovskoy, C. Lin, C. Neu, J. Wood, S. Gollapinni, R. Harr, P. E. Karchin, C. Kottachchi Kankanamge Don, P. Lamichhane, A. Sakharov, D. A. Belknap, L. Borrello, D. Carlsmith, M. Cepeda, S. Dasu, S. Duric, E. Friis, M. Grothe, R. Hall-Wilton, M. Herndon, A. Hervé, P. Klabbers, J. Klukas, A. Lanaro, R. Loveless, A. Mohapatra, I. Ojalvo, T. Perry, G. A. Pierro, G. Polese, I. Ross, T. Sarangi, A. Savin, W. H. Smith, J. Swanson

**Affiliations:** 1 Yerevan Physics Institute, Yerevan, Armenia; 2 Institut für Hochenergiephysik der OeAW, Wien, Austria; 3 National Centre for Particle and High Energy Physics, Minsk, Belarus; 4 Universiteit Antwerpen, Antwerp, Belgium; 5 Vrije Universiteit Brussel, Brussels, Belgium; 6 Université Libre de Bruxelles, Brussels, Belgium; 7 Ghent University, Ghent, Belgium; 8 Université Catholique de Louvain, Louvain-la-Neuve, Belgium; 9 Université de Mons, Mons, Belgium; 10 Centro Brasileiro de Pesquisas Fisicas, Rio de Janeiro, Brazil; 11 Universidade do Estado do Rio de Janeiro, Rio de Janeiro, Brazil; 12 Universidade Estadual Paulista, São Paulo, Brazil; 13 Universidade Federal do ABC, São Paulo, Brazil; 14 Institute for Nuclear Research and Nuclear Energy, Sofia, Bulgaria; 15 University of Sofia, Sofia, Bulgaria; 16 Institute of High Energy Physics, Beijing, China; 17 State Key Laboratory of Nuclear Physics and Technology, Peking University, Beijing, China; 18 Universidad de Los Andes, Bogotá, Colombia; 19 Technical University of Split, Split, Croatia; 20 University of Split, Split, Croatia; 21 Institute Rudjer Boskovic, Zagreb, Croatia; 22 University of Cyprus, Nicosia, Cyprus; 23 Charles University, Prague, Czech Republic; 24 Academy of Scientific Research and Technology of the Arab Republic of Egypt, Egyptian Network of High Energy Physics, Cairo, Egypt; 25 National Institute of Chemical Physics and Biophysics, Tallinn, Estonia; 26 Department of Physics, University of Helsinki, Helsinki, Finland; 27 Helsinki Institute of Physics, Helsinki, Finland; 28 Lappeenranta University of Technology, Lappeenranta, Finland; 29 DSM/IRFU, CEA/Saclay, Gif-sur-Yvette, France; 30 Laboratoire Leprince-Ringuet, Ecole Polytechnique, IN2P3-CNRS, Palaiseau, France; 31 Institut Pluridisciplinaire Hubert Curien, Université de Strasbourg, Université de Haute Alsace Mulhouse, CNRS/IN2P3, Strasbourg, France; 32 Centre de Calcul de l’Institut National de Physique Nucleaire et de Physique des Particules, CNRS/IN2P3, Villeurbanne, France; 33 Institut de Physique Nucléaire de Lyon, Université de Lyon, Université Claude Bernard Lyon 1, CNRS-IN2P3, Villeurbanne, France; 34 Institute of High Energy Physics and Informatization, Tbilisi State University, Tbilisi, Georgia; 35 RWTH Aachen University, I. Physikalisches Institut, Aachen, Germany; 36 RWTH Aachen University, III. Physikalisches Institut A, Aachen, Germany; 37 RWTH Aachen University, III. Physikalisches Institut B, Aachen, Germany; 38 Deutsches Elektronen-Synchrotron, Hamburg, Germany; 39 University of Hamburg, Hamburg, Germany; 40 Institut für Experimentelle Kernphysik, Karlsruhe, Germany; 41 Institute of Nuclear and Particle Physics (INPP), NCSR Demokritos, Aghia Paraskevi, Greece; 42 University of Athens, Athens, Greece; 43 University of Ioánnina, Ioannina, Greece; 44 Wigner Research Centre for Physics, Budapest, Hungary; 45 Institute of Nuclear Research ATOMKI, Debrecen, Hungary; 46 University of Debrecen, Debrecen, Hungary; 47 National Institute of Science Education and Research, Bhubaneswar, India; 48 Panjab University, Chandigarh, India; 49 University of Delhi, Delhi, India; 50 Saha Institute of Nuclear Physics, Kolkata, India; 51 Bhabha Atomic Research Centre, Mumbai, India; 52 Tata Institute of Fundamental Research-EHEP, Mumbai, India; 53 Tata Institute of Fundamental Research-HECR, Mumbai, India; 54 Institute for Research in Fundamental Sciences (IPM), Tehran, Iran; 55 University College Dublin, Dublin, Ireland; 56 INFN Sezione di Bari, Bari, Italy; 57 Università di Bari, Bari, Italy; 58 Politecnico di Bari, Bari, Italy; 59 INFN Sezione di Bologna, Bologna, Italy; 60 Università di Bologna, Bologna, Italy; 61 INFN Sezione di Catania, Catania, Italy; 62 Università di Catania, Catania, Italy; 63 INFN Sezione di Firenze, Florence, Italy; 64 Università di Firenze, Florence, Italy; 65 INFN Laboratori Nazionali di Frascati, Frascati, Italy; 66 INFN Sezione di Genova, Genoa, Italy; 67 Università di Genova, Genoa, Italy; 68 INFN Sezione di Milano-Bicocca, Milan, Italy; 69 Università di Milano-Bicocca, Milan, Italy; 70 INFN Sezione di Napoli, Naples, Italy; 71 Università di Napoli ‘Federico II’, Naples, Italy; 72 Università della Basilicata, Potenza, Italy; 73 Università G. Marconi, Rome, Italy; 74 INFN Sezione di Padova, Padua, Italy; 75 Università di Padova, Padua, Italy; 76 Università di Trento, Trento, Italy; 77 INFN Sezione di Pavia, Pavia, Italy; 78 Università di Pavia, Pavia, Italy; 79 INFN Sezione di Perugia, Perugia, Italy; 80 Università di Perugia, Perugia, Italy; 81 INFN Sezione di Pisa, Pisa, Italy; 82 Università di Pisa, Pisa, Italy; 83 Scuola Normale Superiore di Pisa, Pisa, Italy; 84 INFN Sezione di Roma, Rome, Italy; 85 Università di Roma, Rome, Italy; 86 INFN Sezione di Torino, Turin, Italy; 87 Università di Torino, Turin, Italy; 88 Università del Piemonte Orientale, Novara, Italy; 89 INFN Sezione di Trieste, Trieste, Italy; 90 Università di Trieste, Trieste, Italy; 91 Kangwon National University, Chunchon, Korea; 92 Kyungpook National University, Taegu, Korea; 93 Institute for Universe and Elementary Particles, Chonnam National University, Kwangju, Korea; 94 Korea University, Seoul, Korea; 95 University of Seoul, Seoul, Korea; 96 Sungkyunkwan University, Suwon, Korea; 97 Vilnius University, Vilnius, Lithuania; 98 Centro de Investigacion y de Estudios Avanzados del IPN, Mexico City, Mexico; 99 Universidad Iberoamericana, Mexico City, Mexico; 100 Benemerita Universidad Autonoma de Puebla, Puebla, Mexico; 101 Universidad Autónoma de San Luis Potosí, San Luis Potosí, Mexico; 102 University of Auckland, Auckland, New Zealand; 103 University of Canterbury, Christchurch, New Zealand; 104 National Centre for Physics, Quaid-I-Azam University, Islamabad, Pakistan; 105 National Centre for Nuclear Research, Swierk, Poland; 106 Institute of Experimental Physics, Faculty of Physics, University of Warsaw, Warsaw, Poland; 107 Laboratório de Instrumentaçao e Física Experimental de Partículas, Lisbon, Portugal; 108 Joint Institute for Nuclear Research, Dubna, Russia; 109 Petersburg Nuclear Physics Institute, Gatchina, St. Petersburg, Russia; 110 Institute for Nuclear Research, Moscow, Russia; 111 Institute for Theoretical and Experimental Physics, Moscow, Russia; 112 P.N. Lebedev Physical Institute, Moscow, Russia; 113 Skobeltsyn Institute of Nuclear Physics, Lomonosov Moscow State University, Moscow, Russia; 114 State Research Center of Russian Federation, Institute for High Energy Physics, Protvino, Russia; 115 Faculty of Physics and Vinca Institute of Nuclear Sciences, University of Belgrade, Belgrade, Serbia; 116 Centro de Investigaciones Energéticas Medioambientales y Tecnológicas (CIEMAT), Madrid, Spain; 117 Universidad Autónoma de Madrid, Madrid, Spain; 118 Universidad de Oviedo, Oviedo, Spain; 119 Instituto de Física de Cantabria (IFCA), CSIC-Universidad de Cantabria, Santander, Spain; 120 CERN, European Organization for Nuclear Research, Geneva, Switzerland; 121 Paul Scherrer Institut, Villigen, Switzerland; 122 Institute for Particle Physics, ETH Zurich, Zurich, Switzerland; 123 Universität Zürich, Zurich, Switzerland; 124 National Central University, Chung-Li, Taiwan; 125 National Taiwan University (NTU), Taipei, Taiwan; 126 Chulalongkorn University, Bangkok, Thailand; 127 Cukurova University, Adana, Turkey; 128 Physics Department, Middle East Technical University, Ankara, Turkey; 129 Bogazici University, Istanbul, Turkey; 130 Istanbul Technical University, Istanbul, Turkey; 131 National Scientific Center, Kharkov Institute of Physics and Technology, Kharkiv, Ukraine; 132 University of Bristol, Bristol, UK; 133 Rutherford Appleton Laboratory, Didcot, UK; 134 Imperial College, London, UK; 135 Brunel University, Uxbridge, UK; 136 Baylor University, Waco, USA; 137 The University of Alabama, Tuscaloosa, USA; 138 Boston University, Boston, USA; 139 Brown University, Providence, USA; 140 University of California, Davis, Davis, USA; 141 University of California, Los Angeles, USA; 142 University of California, Riverside, USA; 143 University of California, San Diego, La Jolla, USA; 144 University of California, Santa Barbara, Santa Barbara, USA; 145 California Institute of Technology, Pasadena, USA; 146 Carnegie Mellon University, Pittsburgh, USA; 147 University of Colorado at Boulder, Boulder, USA; 148 Cornell University, Ithaca, USA; 149 Fairfield University, Fairfield, USA; 150 Fermi National Accelerator Laboratory, Batavia, USA; 151 University of Florida, Gainesville, USA; 152 Florida International University, Miami, USA; 153 Florida State University, Tallahassee, USA; 154 Florida Institute of Technology, Melbourne, USA; 155 University of Illinois at Chicago (UIC), Chicago, USA; 156 The University of Iowa, Iowa City, USA; 157 Johns Hopkins University, Baltimore, USA; 158 The University of Kansas, Lawrence, USA; 159 Kansas State University, Manhattan, USA; 160 Lawrence Livermore National Laboratory, Livermore, USA; 161 University of Maryland, College Park, USA; 162 Massachusetts Institute of Technology, Cambridge, USA; 163 University of Minnesota, Minneapolis, USA; 164 University of Mississippi, Oxford, USA; 165 University of Nebraska-Lincoln, Lincoln, USA; 166 State University of New York at Buffalo, Buffalo, USA; 167 Northeastern University, Boston, USA; 168 Northwestern University, Evanston, USA; 169 University of Notre Dame, Notre Dame, USA; 170 The Ohio State University, Columbus, USA; 171 Princeton University, Princeton, USA; 172 University of Puerto Rico, Mayaguez, USA; 173 Purdue University, West Lafayette, USA; 174 Purdue University Calumet, Hammond, USA; 175 Rice University, Houston, USA; 176 University of Rochester, Rochester, USA; 177 The Rockefeller University, New York, USA; 178 Rutgers, The State University of New Jersey, Piscataway, USA; 179 University of Tennessee, Knoxville, USA; 180 Texas A&M University, College Station, USA; 181 Texas Tech University, Lubbock, USA; 182 Vanderbilt University, Nashville, USA; 183 University of Virginia, Charlottesville, USA; 184 Wayne State University, Detroit, USA; 185 University of Wisconsin, Madison, USA; 186 CERN, Geneva, Switzerland

## Abstract

A study of color coherence effects in pp collisions at a center-of-mass energy of 7$\,\text {TeV}$ is presented. The data used in the analysis were collected in 2010 with the CMS detector at the LHC and correspond to an integrated luminosity of 36 pb$^{-1}$. Events are selected that contain at least three jets and where the two jets with the largest transverse momentum exhibit a back-to-back topology. The measured angular correlation between the second- and third-leading jet is shown to be sensitive to color coherence effects, and is compared to the predictions of Monte Carlo models with various implementations of color coherence. None of the models describe the data satisfactorily.

## Introduction

An important feature of the color interaction in quantum chromodynamics (QCD) is that the outgoing partons produced in the hard interaction continue to interfere with each other during their fragmentation phase. This phenomenon, called *color coherence*, manifests itself by the relative abundance of soft radiation in the region between the color connected final-state partons and the suppression of soft radiation elsewhere.

Color coherence phenomena were initially observed in $\mathrm {e}^+\mathrm {e}^-$ collisions by several experiments at PETRA, PEP and LEP [1–8]. These experiments showed the coherence effect in $\mathrm {e}^+\mathrm {e}^- \rightarrow \text {q}\bar{\mathrm{q}}\text {g}$ three-jet events through the suppression of particle production in the region between the quark and antiquark jets.

In hadron collisions, in addition to the color connection between the final-state partons, the color connection between the outgoing partons and the incoming partons must be considered. The Tevatron experiments CDF and D0 have both reported evidence for color coherence effects in measurements of the spatial correlations between neighboring jets [9, 10]. These correlations were not well reproduced by Monte Carlo (MC) simulations that use incoherent parton shower models. However, the data were successfully described by simulations that include color coherence effects through the ordering of the parton emission angles [11].

The technique originally developed by the Tevatron experiments is used to study color coherence effects in pp collisions at $\sqrt{s}=7$ $\,\text {TeV}$ with the Compact Muon Solenoid (CMS) detector. Events with at least three jets (called three-jet events) are selected, and these jets are ordered by their transverse momenta $p_{\mathrm {T1}}>p_{\mathrm {T2}}>p_{\mathrm {T3}}$ with respect to the beam direction. We measure the angular correlation between the second and third jet to probe the effects of color coherence.

The CMS detector has a right-handed coordinate system with its origin at the center of the detector. The $z$ axis points along the direction of the counterclockwise beam, $\phi$ is the azimuthal angle in the transverse plane perpendicular to the beam, and $\theta$ is the polar angle relative to the $z$ axis. The pseudorapidity of the $i$th jet is denoted by $\eta _i=-\ln [\tan (\theta _i/2)]$ and its azimuthal angle by $\phi _i$.

**Fig. 1:**
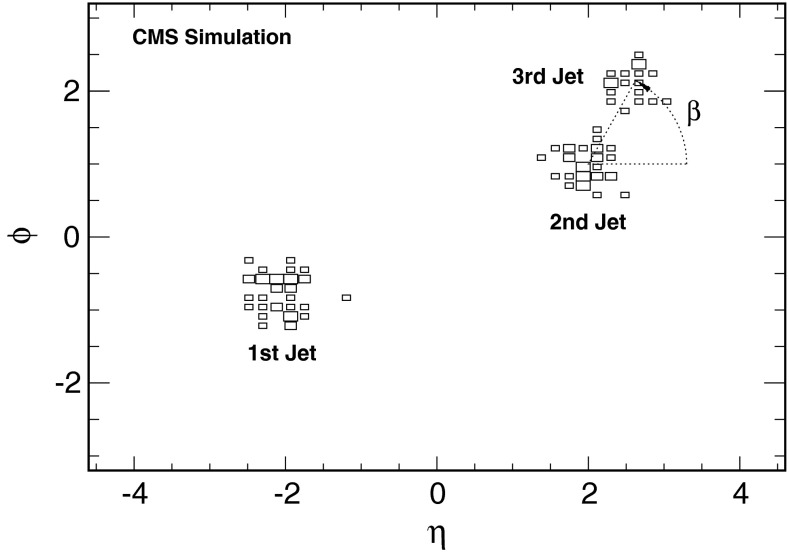
Visualization of the observable $\beta$ in ($\eta ,\phi$) space using a simulated three-jet event. The sizes of the *rectangular boxes* are proportional to the particle energies

The measured observable $\beta$ [10] is defined as the azimuthal angle of the third jet with respect to the second jet in ($\eta ,\phi$) space as shown in Fig. 1. Implicitly, this can be expressed by

1 $$\begin{aligned} \tan \beta =\frac{|\varDelta \phi _{23} |}{\varDelta \eta _{23}}, \end{aligned}$$

where $\varDelta \phi _{23}=\phi _3-\phi _2$ (defined so that $-\pi \le \varDelta \phi _{23}\le \pi$), $\varDelta \eta _{23} = {{\mathrm{sign}}}(\eta _2) \cdot (\eta _3 - \eta _2)$, and $0 \le \beta \le \pi$. The absolute value of $\varDelta \phi _{23}$ in Eq. 1 and the sign of the pseudorapidity of the second jet, ${{\mathrm{sign}}}(\eta _2)$, in the definition of $\varDelta \eta _{23}$ are introduced to map symmetric configurations around $\varDelta \phi _{23} = 0$ or $\eta = 0$ onto the same $\beta$ value. For $\varDelta \phi _{23} = 0$, $\beta$ is defined to be zero or $\pi$ depending on the sign of $\varDelta \eta _{23}$ being positive or negative. In the case of $\varDelta \eta _{23} = 0$, which cannot happen simultaneously with $\varDelta \phi _{23} = 0$, $\beta$ is defined to equal $\pi /2$.

In a naive leading-order model the two partons are produced back-to-back in the transverse plane. One of the two partons may radiate a third parton. In the absence of color coherence effects there is no preferred direction of emission of this third parton around the radiating parton. In contrast, when color coherence effects are present, the third parton will tend to lie in the event plane defined by the emitting parton and the beam axis. Therefore, in the presence of color coherence, the third jet population along the event plane (in particular near $\beta \approx 0$) will be enhanced and out of the plane ($\beta \approx \pi /2$) will be suppressed. The color coherence effects are expected to become stronger in the region between the second jet and the remnant when the angle between them becomes smaller. Therefore the study of the $\beta$ variable is performed in two situations: when the second jet is rather central ($|\eta _2 | \le 0.8$) and when the second jet is more forward ($0.8 < |\eta _2 | \le 2.5$).

The aims of this paper are

- To measure the $\beta$ distributions, normalized to the total number of events in each region, as a function of $\beta$ separately in the central ($|\eta _2 | \le 0.8$) and forward region ($0.8<|\eta _2 | \le 2.5$):
  2 $$\begin{aligned} F_{\eta _2, i}(\beta ) = \frac{N_{\eta , i}}{N_{\eta }}, \end{aligned}$$
  where $N_{\eta }$ is the total number of events in the $\eta _2$ region, $N_{\eta , i}$ the number of events in the given $i$th $\beta$ bin of the $\eta _2$ region. The choice of this normalization significantly reduces the impact of experimental systematic uncertainties such as the uncertainty in the luminosity.
- To gauge the sensitivity of the variable $\beta$ to color coherence effects.
- To compare our measurements to the predictions of MC event generators with various implementations of color coherence.

## The CMS detector

A detailed description of the CMS experiment can be found elsewhere [12]; so here we describe the detector systems most relevant to the present analysis. The central feature of the CMS apparatus is a superconducting solenoid of 6 m internal diameter, providing a magnetic field of 3.8 T. Within the field volume, a silicon pixel and strip tracker, a lead tungstate crystal electromagnetic calorimeter and a brass/scintillator hadron calorimeter (HCAL) are installed. The central tracking system provides coverage up to $|\eta |= 2.5$ in pseudorapidity and the calorimeters up to $|\eta |=3.0$. An iron and quartz-fiber Cherenkov forward hadron calorimeter (HF) covers the pseudorapidity range $3.0<|\eta |<5.0$.

## Event selection

The CMS detector records events using a two-level trigger system consisting of a hardware-based level-1 (L1) trigger and a software-based high-level trigger (HLT). For this study, single jet triggers that reconstruct jets from calorimeter energy deposits at L1 and HLT are used to select events based on different $p_{\mathrm {T}}$ jet thresholds. Five different triggers with $p_{\mathrm {T}}$ thresholds of 30, 50, 70, 100, and 140$\,\text {GeV}$ are used to select the events. The triggers were prescaled during the 2010 run when the associated rate exceeded the allocated band width except the highest-threshold one. Therefore, the events are split into five different bins in $p_{\mathrm {T1}}$ with each bin containing the events collected during a period when the appropriate trigger was not prescaled. Each bin starts at $p_{\mathrm {T}\text {min}}$ defined in such a way that the associated trigger efficiency exceeds 99 %. Table 2 lists the binning in $p_{\mathrm {T1}}$, and, for each bin, it gives the associated trigger, the number of selected events, and the integrated luminosity for the period during which the given trigger was not prescaled.

Jets are reconstructed with the anti-$k_{\mathrm {T}}$ algorithm [13], which is implemented in the FastJet package [14] using a distance parameter $R = 0.5$, from a list of particle candidates reconstructed using the particle-flow (PF) algorithm. This PF algorithm [15] reconstructs all particle candidates in each event using an optimized combination of information from all CMS subdetector systems: muons, electrons (with associated bremsstrahlung photons), photons (unconverted and converted), and charged/neutral hadrons. The four-vectors of the

neutral particles are computed by assuming that they come from the primary vertex, which is defined as the vertex with the highest sum of transverse momenta of all reconstructed tracks pointing to it. The reconstructed jet energy $E$ is defined as the scalar sum of the energies of the constituents, and the jet momentum ${{\varvec{p}}}$ is the vector sum of the momenta of the constituents. The jet transverse momentum $p_{\mathrm {T}}$ is the component of ${{\varvec{p}}}$ perpendicular to the beam. The $E$ and ${{\varvec{p}}}$ values of a reconstructed jet are further corrected for the response of the detector, which is obtained from MC simulations, test beam results, and pp collision data [16, 17]. The corrections account for the presence of multiple pp collisions in the same or adjacent bunch crossings (pileup interactions) using the jet area method [18].

Events are required to have a primary vertex reconstructed within 24 cm of the detector center along the beam line [19]. Additional selection criteria are applied to each event to remove any spurious jet-like features originating from isolated noise patterns in certain HCAL regions [20]. Events having at least three jets with $p_{\mathrm {T}} >30$ $\,\text {GeV}$ are selected. The pseudorapidity of the two leading jets must be within $|\eta _{1} |,|\eta _{2} |\le 2.5$, while for the third jet no constraints are applied in order to avoid a bias in the $\beta$ measurement.

To further reduce the background from misidentified jets, i.e., jets resulting from noise in the electromagnetic, hadron and/or hadron forward calorimeters, a set of tight identification criteria are applied: each jet should contain at least two particles, one of which is a charged hadron, and the jet energy fraction carried by neutral hadrons, photons, muons, and electrons should be less than 90 %. With these criteria the contamination of the sample with misidentified jets is suppressed to a level less than 1 % [15].

The dijet invariant mass of the two leading jets, $M_{12}$, is required to exceed 220 $\,\text {GeV}$ to ensure a back-to-back configuration. With this requirement more than 98 % of the events have $|\varDelta \phi _{12}-\pi |<1$. Finally the distance in the ($\eta ,\phi$) space between the second and third jets is constrained to be $0.5<\varDelta R_{23} = \sqrt{(\varDelta \eta _{23})^2 + (\varDelta \phi _{23})^2}<1.5$ in order to ensure a three-jet topology where the third jet is closer to the second jet.

**Fig. 2:**
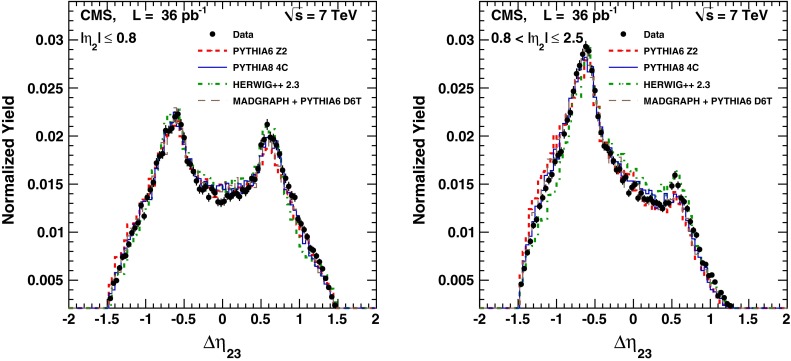
Observed $\varDelta \eta _{23}$ distributions, corrected for detector effects, compared to MC predictions by pythia  6, pythia  8, herwig++, and MadGraph  + pythia  6. The MC samples are normalized to the total number of events in data

**Fig. 3:**
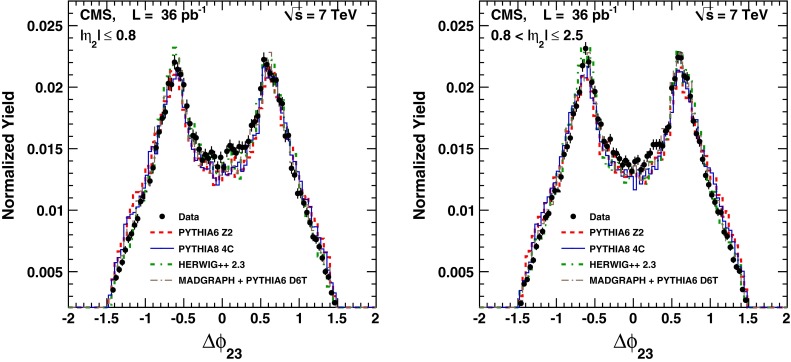
Observed $\varDelta \phi _{23}$ distributions, corrected for detector effects, compared to MC predictions by pythia  6, pythia  8, herwig++, and MadGraph  + pythia  6. The MC samples are normalized to the total number of events in data

The selections used in the analysis are summarized in Table 1. The numbers of events passing the selection criteria in each $p_{\mathrm {T1}}$ bin are summarized in Table 2. The measured $\varDelta \eta _{23}$ and $\varDelta \phi _{23}$ distributions are compared to various MC models in Figs. 2 and 3. In general a reasonable agreement is observed with the different models. A study of the amount of energy collected by the HF detector indicated that there is no diffractive component in the data sample.

**Table 1:** Summary of the event selection

Selection criteria	
$p_{\mathrm {T1}}>100$ $\,\text {GeV}$, $p_{\mathrm {T3}}>30$ $\,\text {GeV}$	
$$|\eta _1 |,|\eta _2 | \le 2.5$$	
$M_{12}>220$ $\,\text {GeV}$	
$$0.5<\varDelta R_{23}<1.5$$

**Table 2:** The binning in $p_{\mathrm {T1}}$ and, for each bin, the associated trigger, the integrated luminosity for the period during which the given trigger was not prescaled, and the number of selected events. The selection criteria are described in Table 1

$p_{\mathrm {T1}}$ bin edges	Trigger online	$\mathcal {L} _\mathrm{int}$	Number of events		
($\text {GeV}$ )	threshold ($\text {GeV}$ )	(pb$^{-1}$)			
			Total	$|\eta _2 | \le 0.8$	$0.8<|\eta _2 | \le 2.5$
100–120	30	0.35	4511	1671	2840
120–160	50	4.5	67 086	27 069	40 017
160–200	70	9.2	50 071	23 055	27 016
200–250	100	20	39 464	18 987	20 477
${>}250$	140	36	31 999	16 728	15 271
All			193 131	87 510	105 621

## Monte Carlo models

The reconstructed jets are compared to the predictions of four different Monte Carlo generators that simulate jet production in pp collisions at $\sqrt{s} = 7\,\text {TeV}$. The numbers of events for all generator samples is much higher than the number of collected data events so the statistical uncertainties in the MC predictions are not visible in the figures.

The pythia [21] (version 6.422) event generator uses leading-order (LO) matrix elements to generate the 2 $\rightarrow$ 2 hard process in perturbative QCD (pQCD) and the parton shower (PS) model to simulate higher-order processes [22–24]. The PS model gives a good description of parton emission when the emitted partons are close in phase space. Events are generated with the Z2 tune for the underlying event. This Z2 tune is identical to the Z1 tune described in Ref. [25], except that Z2 uses the CTEQ6L1 [26] parton distribution functions (PDFs) of the proton in which the parton showers are ordered in $p_{\mathrm {T}}$. The hadronization is simulated using the Lund string model [27, 28]. The older D6T tune [29–31], where parton showers are ordered in $Q^2$, is considered for comparison. The D6T tune was designed to describe the lower-energy results of UA5 and CDF. The color coherence effects are implemented in pythia  6 by means of an angular ordering algorithm where the effects can be switched on and off via the steering parameters MSTP(67) and MSTJ(50), which control the initial-state and the final-state showers, respectively.

The pythia  8 [32] (version 8.145) event generator, used with tune 4C [33], orders the parton showers in $p_{\mathrm {T}}$ and models the underlying event using the multiple-parton interaction model from pythia  6 including initial- and final-state QCD radiation. The color coherence effects are implemented in a similar manner as for the $p_{\mathrm {T}}$-ordered showers in pythia  6.

The herwig++ [11, 34] (version 2.4.2) event generator takes LO matrix elements and simulates parton showers using the coherent branching algorithm with angular ordering of showers. The cluster hadronization model [35] is used in the formation of hadrons from the quarks and gluons produced in the parton shower. The underlying event is simulated using the eikonal multiple partonic scattering model [36]. The color coherence effects are implemented by the angular ordering of emissions in the parton shower using the coherent branching algorithm [37].

The MadGraph  4 [38] (version 2.24) event generator is interfaced with pythia  6 for the parton showering and the hadronization using the D6T tune and uses fixed-order matrix element calculations for the multiparton topologies. From two to four partons are considered in the final state. The color coherence for the hard jets at leading order comes from the exact QCD color amplitudes in the model. The $k_{\mathrm {T}}$ MLM matching scheme [39] applied with a matching parameter of 60$\,\text {GeV}$ avoids double-counting between the partons from MadGraph and the PS.

## Measurement of the normalized $\beta$ distribution and systematic uncertainties

The measurement of the $\beta$ distribution is performed in two regions defined by the pseudorapidity of the second jet: the central region $|\eta _2 | \le 0.8$ and the forward region $0.8<|\eta _2 | \le 2.5$. The angular correlation effects considered in this analysis appear to have a reduced sensitivity to the transverse momentum of the leading jet $p_{\mathrm {T1}}$. Consequently different $p_{\mathrm {T1}}$ bins are merged into one single bin.

The $\beta$ distribution in a given $\eta _2$ region is obtained as a sum of the events weighted by the luminosity collected by the trigger used in the associated $p_{\mathrm {T1}}$ bin. In case of MC samples the $\beta$ distribution is obtained by summing together the events weighted by their generation level weight in a given $\eta _2$ region. The normalized $\beta$ distribution is then obtained by dividing the weighted number of events in a given bin of $\beta$ by the total weighted number of events in the given $\eta _2$ region.

In order to correct for the smearing effects induced by the detector resolution, an unfolding procedure is performed using the response matrices obtained from MC event generators. For this purpose the events generated with the MC programs (pythia  6, pythia  8, MadGraph  + pythia  6, and herwig++) are processed through a full CMS detector simulation package based on geant 4 [40].

Particle-level jets are built from the four-vectors of the MC generated particles with hadronization, but without detector effects. These jets are obtained using the same jet algorithm as for the reconstructed events. The resolutions in $\varDelta \eta _{23}$ and $\varDelta \phi _{23}$ are found to be of the order of 0.005 to 0.01, depending on the transverse momentum and pseudorapidity of the jets.

An iterative Bayesian unfolding technique [41] implemented in the RooUnfold package [42] is used to derive the unfolding corrections to the measured $\beta$ distributions from the detector effects. The response matrix used to unfold the data is built using herwig++. The impact of the unfolding on the normalized distributions is typically of the order of 1 %.

Most of the systematic effects cancel out in the normalized $\beta$ distribution, but the residual influence of several sources of systematic uncertainty has been considered:

- The jet energy scale uncertainty is evaluated varying the jet response by 2.5–5 %, depending on the $\eta$ and $p_{\mathrm {T}}$ of the jets [43]. The impact of this source of systematic uncertainties is below $1~\%$.
- The jet energy and angular resolutions are accounted for by varying them by ${\pm }10~\%$ [44] and rebuilding the response matrices for the unfolding accordingly. The observed impact from both sources is in the range of 0.4–0.6 %.
- The uncertainty due to the unfolding procedure is estimated by the dependence of the response matrix on the choice of MC generator, Alternative response matrices are built using alternative generators: pythia  6, pythia  8 and MadGraph  + pythia  6. The observed effect is of the order of 0.5 %.

The measurement is found to be insensitive to the number of pileup interactions within statistical fluctuations. In the data corresponding to this analysis the average number of pileup events per bunch crossing was around two. The total systematic uncertainties for each bin are about 2 %, and a list of the major uncertainties is summarized in Table 3. Each systematic source was found to be fully correlated between $\beta$ and $\eta _2$ bins [43, 44]. However, the various systematic sources are uncorrelated among themselves.

**Table 3:** Typical systematic and statistical uncertainties in the normalized $\beta$ spectrum and the statistical errors

Uncertainty sources	$|\eta _2 | \le 0.8$	$0.8<|\eta _2 | \le 2.5$
Jet energy scale (JES)	1.0 %	1.0 %
Jet energy resolution (JER)	0.4 %	0.5 %
Jet angular resolution (JAR)	0.5 %	0.6 %
Physics model (PM) used in unfolding	0.6 %	0.7 %
Statistical uncertainty	4.0 %	3.7 %

## Results

**Fig. 4:**
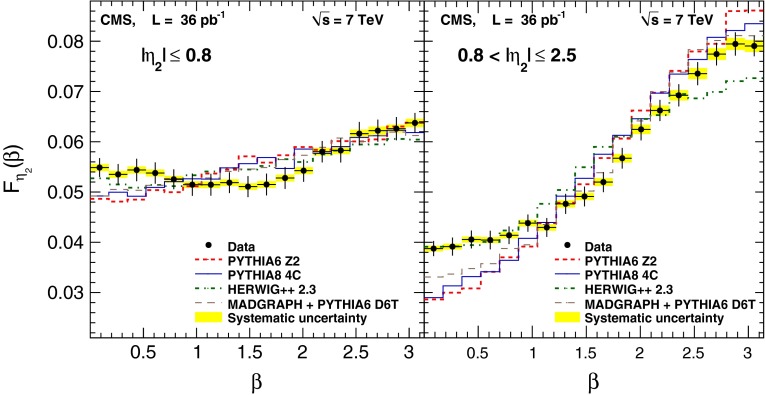
Observed $\beta$ distributions for the data, corrected for detector effects, and for the MC generators (pythia  6, pythia  8, herwig++, and MadGraph  + pythia  6) in the central ($|\eta _{2} | \le 0.8$) and forward ($0.8 < |\eta _2 | \le 2.5$) regions. The *error bars* show the statistical uncertainties, while the *yellow shaded bands* correspond to the combined systematic uncertainty

The unfolded $\beta$ distributions are shown in Fig. 4 together with the predictions from the various MC models for the central ($|\eta _2 | \le 0.8$) and forward ($0.8 < |\eta _2 | \le 2.5$) regions.

The values of the unfolded $\beta$ distributions and their uncertainties are presented in Tables 4 and 5.

**Table 4:** The unfolded $\beta$ distributions and their uncertainties for the central region $|\eta _2 | \le 0.8$. All uncertainties are symmetric and given in percent ($\%$)

$\beta$ (degree)	$F_{\eta _2}(\beta )$	$\sigma _\mathrm{{Stat}}$	$\sigma _\mathrm{{JES}}$	$\sigma _\mathrm{{JER}}$	$\sigma _\mathrm{{JAR}}$	$\sigma _\mathrm{{PM}}$	$\sigma _\mathrm{{Syst}}$
0–10	0.0549	3.5	1.0	0.3	0.4	0.6	1.3
10–20	0.0535	3.9	1.1	0.4	0.6	0.6	1.4
20–30	0.0544	4.2	0.5	0.5	0.3	0.6	1.0
30–40	0.0538	4.0	1.1	0.2	0.3	0.6	1.3
40–50	0.0525	3.8	0.5	0.5	0.5	0.6	1.1
50–60	0.0515	4.4	0.6	0.6	0.7	0.6	1.3
60–70	0.0515	4.3	0.6	0.4	0.6	0.6	1.1
70–80	0.0519	4.1	0.5	0.3	0.4	0.6	0.9
80–90	0.0511	4.2	0.4	0.4	0.5	0.6	1.0
90–100	0.0515	4.3	0.5	0.3	0.2	0.6	0.9
100–110	0.0528	4.3	0.5	0.4	0.5	0.6	1.0
110–120	0.0543	4.3	0.6	0.6	0.3	0.6	1.1
120–130	0.0580	4.1	1.2	0.5	0.4	0.6	1.5
130–140	0.0583	3.7	0.5	0.6	0.3	0.6	1.0
140–150	0.0616	4.2	0.6	0.5	0.5	0.6	1.1
150–160	0.0622	3.9	0.9	0.6	0.5	0.6	1.3
160–170	0.0626	3.6	0.7	0.5	0.6	0.6	1.2
170–180	0.0638	3.2	0.5	0.7	0.6	0.6	1.2

**Table 5:** The unfolded $\beta$ distributions and their uncertainties for the forward region $0.8<|\eta _2 | \le 2.5$. All uncertainties are symmetric and given in percent ($\%$)

$\beta$ (degree)	$F_{\eta _2}(\beta )$	$\sigma _\mathrm{{Stat}}$	$\sigma _\mathrm{{JES}}$	$\sigma _\mathrm{{JER}}$	$\sigma _\mathrm{{JAR}}$	$\sigma _\mathrm{{PM}}$	$\sigma _\mathrm{{Syst}}$
0–10	0.0388	3.9	1.6	0.5	0.5	0.7	1.9
10–20	0.0391	4.6	0.6	0.5	0.6	0.7	1.2
20–30	0.0406	4.4	0.7	0.4	0.5	0.7	1.2
30–40	0.0404	4.6	0.5	0.4	0.5	0.7	1.1
40–50	0.0414	4.2	0.6	0.5	0.5	0.7	1.2
50–60	0.0438	3.9	0.7	0.4	0.4	0.7	1.1
60–70	0.0430	4.4	0.8	0.5	0.6	0.7	1.3
70–80	0.0476	4.2	0.5	0.5	0.6	0.7	1.2
80–90	0.0491	4.0	1.2	0.4	0.5	0.7	1.5
90–100	0.0520	3.9	0.8	0.5	0.4	0.7	1.2
100–110	0.0567	3.6	0.8	0.5	0.5	0.7	1.3
110–120	0.0625	3.5	0.7	0.5	0.5	0.7	1.2
120–130	0.0662	3.2	0.8	0.5	0.6	0.7	1.3
130–140	0.0692	3.2	0.7	0.4	0.6	0.7	1.2
140–150	0.0736	3.1	0.6	0.6	0.5	0.7	1.2
150–160	0.0774	2.9	0.7	0.4	0.6	0.7	1.2
160–170	0.0795	2.9	0.8	0.5	0.5	0.7	1.3
170–180	0.0791	2.6	0.8	0.6	0.5	0.7	1.3

**Fig. 5:**
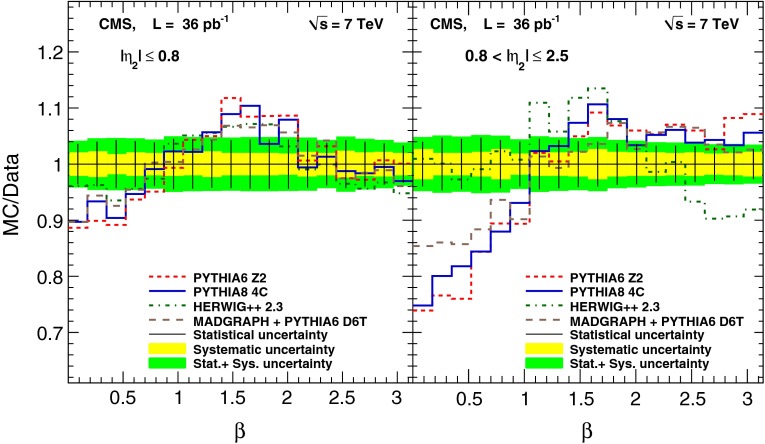
The ratio of the various MC predictions to the measured $\beta$ distribution. The *error bars* show the statistical uncertainty of the data. The *yellow band* represents the systematic uncertainty, while the *green band* represents the total uncertainty

The ratios of the various MC predictions to the measured $\beta$ distributions are shown in Fig. 5. The data exhibit a clear enhancement of events compared to the pythia and MadGraph generators near the event plane ($\beta = 0$) and a suppression in the transverse plane ($\beta = \pi /2$). The $\chi ^2$ comparisons of data with MC simulation, taking into account the statistical and systematic correlations between different data points, are shown separately for the central and forward regions in Table 6. The number of degrees of freedom (NDF) is 17, which is the number of bins minus one to account for the constraint imposed by the normalization.

**Table 6:** Values of $\chi ^2$ for comparisons of the $\beta$ distribution for the data with the predictions of various MC generators. The number of degrees of freedom for both regions is 17

MC event generator	$\chi ^2$/NDF	
	$|\eta _2 | \le 0.8$	$0.8<|\eta _2 | \le 2.5$
pythia 6 Z2	2.5	8.1
pythia 8 4C	1.7	6.4
herwig++ 2.3	1.2	3.5
MadGraph + pythia 6	1.6	3.3

None of the models used in the analysis describes the data satisfactorily. Even though pythia  6 was adjusted with the Tevatron data, it fails to describe the LHC data since the $\chi ^2$/NDF is large. No significant difference is observed between the tunes D6T and Z2. The pythia  8 tune 4C generator describes the data better than pythia  6 over the entire phase space, but the disagreement in the forward region is not negligible. The herwig++ event generator describes the data better than the other MC generators in the central region, but the agreement is poor in the forward region. Finally, when MadGraph is used with the exact $2 \rightarrow 3$ matrix element calculations at LO, the global description of the data is improved with respect to pythia  6 alone.

**Fig. 6:**
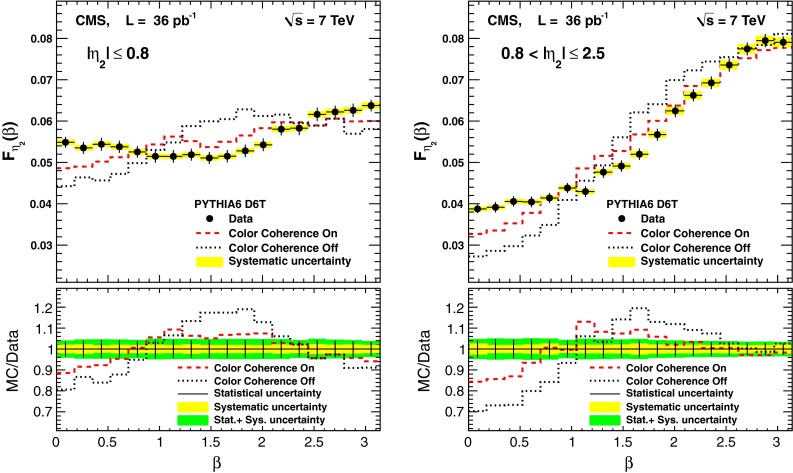
The MC predictions for the $\beta$ distribution from pythia  6, with and without color coherence effects in the first branching of the initial- and final-state showers, compared to the measurement. The *error bars* show the uncorrelated statistical uncertainty of the data. The *yellow band* represents the systematic uncertainty, while the *green band* represents the total uncertainty

The impact of the color coherence effects is studied by switching them on and off for the first emission in the initial- and final-state showers in pythia  6. One can observe in Fig. 6 that the agreement between the data and the simulation deteriorates when the color coherence effects in the MC events are suppressed. More quantitatively, the $\chi ^2$ divided by the number of degrees of freedom increases up to 7.7 in the central region and 11.5 in the forward region. The first emission in the initial- and final-state showers contributes roughly the same order. Using pythia, it has been verified that the impact of the non-perturbative component of the QCD calculation (hadronization and underlying event) is negligible for this analysis. One conclusion from this pythia study, as shown Fig. 6, is that the data clearly support larger color coherence effects than in present MC implementations.

## Summary

Color coherence effects in multijet events have been studied in a sample of pp collisions corresponding to an integrated luminosity of 36 pb$^{-1}$ , collected with the CMS detector at $\sqrt{s} = 7\,\text {TeV}$. Distributions of the variable $\beta$, which was previously used in similar analyses at the Tevatron, are used to measure the angular correlation between the second and third jets in transverse-momentum order, in the pseudorapidity and azimuthal angle space. The measurements, unfolded for detector effects, are compared to the predictions of the MC event generators pythia  6, pythia  8, herwig++, and MadGraph  + pythia  6 in the central and forward rapidity regions. We have shown that the variable $\beta$ is sensitive to color coherence effects, and insensitive to the hadronization and underlying event. It is necessary to implement the color coherence effects in MC simulations to better describe the data. Although the MC models in the analysis include this effect by default, none of them describes the data satisfactorily for all $\beta$ values. The pythia  6 expectations predict weaker color coherence effects than those observed, while pythia  8 exhibits a better agreement with the data. The MadGraph MC generator, which uses the exact $2 \rightarrow 3$ matrix element calculations at LO matched to pythia  6 for parton showering, improves the agreement with data with respect to pythia  6 alone, while herwig++ describes the data in the central region better than the other MC generators but shows discrepancies in the forward region.
